# Investigation on the Fatigue Crack Propagation of Medium-Entropy Alloys with Heterogeneous Microstructures

**DOI:** 10.3390/ma15176081

**Published:** 2022-09-01

**Authors:** Yang Liu, Ping Jiang, Guihua Duan, Jing Wang, Lingling Zhou, Jijia Xie

**Affiliations:** 1 State Key Laboratory of Nonlinear Mechanics, Institute of Mechanics, Chinese Academy of Sciences, Beijing 100190, China; 2 School of Engineering Science, University of Chinese Academy of Sciences, Beijing 100049, China

**Keywords:** heterogeneous microstructure, MEA, recrystallization annealing, fatigue crack propagation threshold, fatigue crack growth path, cyclic plastic zone

## Abstract

The behavior and the mechanism of fatigue crack propagation in CrCoNi medium-entropy alloys (MEAs) with heterogeneous microstructures were investigated in this paper. After cold-rolling and recrystallization annealing at different temperatures and times, five sets of heterostructured specimens were acquired with different recrystallization levels. Then, the structure characterizations of these five sets of specimens were carried out by nanoindentation testing and electron back-scatter diffraction (EBSD) mapping. Finally, the fatigue crack propagation tests were conducted on single edge crack specimens of these different heterogeneous microstructures. The experimental results indicate that the crack propagation rates of specimens with partial recrystallization microstructures are higher than those with complete recrystallization microstructures, and the effect on fatigue crack thresholds of these specimens is the opposite. The fatigue cracks grow along the slip planes or twin boundaries in recrystallization grains (RGs), which induced crack deflections and the roughness-induced crack closure effect. For this reason, the area percentage of recrystallization and the grain size of RGs have a great effect on the value of the fatigue crack growth threshold.

## 1. Introduction

Alloys with heterogeneous microstructures overcome the trade-off between the strength and ductility, and they have become an important category of structural materials. Wu and Zhu [1,2] pointed out that heterogeneous materials consist of domains with a dramatic difference in strength. These kinds of heterogeneous materials include gradient structures, heterogeneous lamella structures, bimodal structures, dual-phase steel, etc. In general, there are related soft domains and hard domains in heterogeneous materials [1,2,3,4]. When these materials deform in plasticity, the deformation of the soft domain is constrained by the hard domain. This constraint induces a pile-up of geometrically necessary dislocations at the interfaces of the soft domains and hard domains, which provides the extra hetero-deformation-induced (HDI) hardening for a superior combination of strength and ductility. Therefore, these materials may be applied to the fields of transportation vehicles and energy to reduce the weight of the structures and ensure the safety of the structures under impact loads. In addition, heterostructured materials can be processed using current industrial facilities, making them conducive to industrial production at low costs [5]. This heterostructured treatment provides a new idea for the development of structural materials, making it possible for even low-strength metals or alloys, such as pure Ti [6], to have a good combination of the high tensile strength of an ultrafine-grained metal and the ductility of a coarse-grained metal. This means that, currently, a heterostructured treatment may be a potential method to improve the performance of existing structural materials.

However, the investigations on the fatigue properties of heterostructured materials and on the quantitative characterizations of the heterostructured levels of the heterostructured materials are still limited. For the heterogeneity of the microstructures, the fatigue damage mechanism of the heterogeneous materials is not immediately clear.

According to traditional points, a metal’s fatigue limit is proportional to its tensile strength or hardness [7,8,9,10], which means that the higher the strength or hardness, the higher the fatigue limit. This is consistent with the results of studies on heterostructured materials with a gradient structure [11,12,13,14,15]. Through surface nanostructuring, such as surface mechanical attrition treatments and surface mechanical grinding treatments, the strength of the surface layer increases with the increase in residual compressive stress, which induces a higher fatigue limit and a longer fatigue life. However, for heterostructured materials with mixed soft zones and hard zones, whether the heterostructured microstructure can improve the material’s fatigue limit is a question.

Liu et al. [16] investigated the fatigue behavior of three groups of Cu-5at%Al specimens with cold-rolled and partially recrystallized and fully recrystallized microstructures. They found that the fatigue limits of the partially recrystallized specimens remained almost constant. For this type of heterostructured material, fatigue cracks might initiate from the soft zones, such as the recrystallized areas. This means that the fatigue limit is controlled by the strength of the soft zones, rather than by the overall strength of the specimen.

Another doubtful point relates to the fatigue crack propagation of heterostructured materials. In general, for uniform materials, coarse-grain specimens have a higher fatigue crack propagation threshold and a lower crack propagation rate [17,18]. However, in the investigation of the near-threshold fatigue propagation in a Ti-6Al-4V alloy of harmonic structure, Kikuchi et al. [19] found that, although it had a higher yield strength and similar elongation to the coarse-grain specimens, the fatigue crack propagation threshold was not dependent on the volume fraction of the fine-grained structure. This means that for heterostructured materials with mixed soft zones and hard zones, it is not known whether their superior combinations of strength and ductility would induce lower or higher levels of fatigue crack resistance.

The objective of this investigation is to study the fatigue crack growth mechanism of CrCoNi MEAs with different heterogeneous microstructures. Scanning electron microscopy (SEM), electron back-scatter diffraction (EBSD), and a nanoindentor were used to characterize the microstructures, the fracture surfaces of the fatigue samples after break, and the crack growth mechanisms along the crack paths.

## 2. Materials and Methods

The experimental material is an equimolar CrCoNi MEA with the actual composition of 33.4Cr-32.3Co-34.3Ni (atomic percent). After being homogenized at 1473 K for 12 h, the original ingot was hot-forged into slabs of 12 mm in thickness, and, finally, cold-rolled into sheets of about 1.5 mm thickness, with a reduction in thickness of 87.5%. For producing specimens with different heterogeneous microstructures, the cold-rolled sheets were annealed at 873 K for 5, 15, 30, and 60 min and named HS1, HS2, HS3, and HS4, respectively. In addition, some cold-rolled sheets were annealed at 1073 K for 60 min as reference specimens with a fully recrystallized microstructure, which were named HS5.

**Figure 1:**
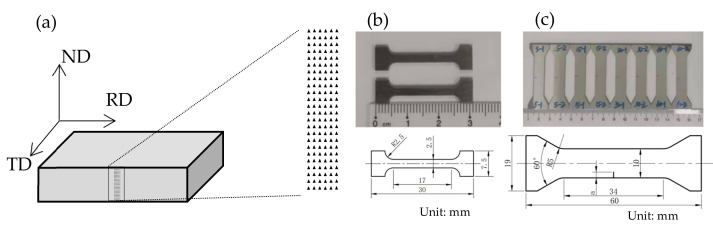
Diagram of the specimens for microstructure characterization and mechanical testing. (**a**) Section for EBSD and nanoindent measurements. (**b**) Tensile specimens. (**c**) Single edge crack tension (SET) specimens for fatigue crack propagation testing.

After the annealing treatment, the plates with different heterogeneous microstructures were cut by wire electrical discharge machining into specimens with dimensions of 10 mm (length) × 5 mm (width). As shown in Figure 1, the TD section, which is perpendicular to the TD direction, was mechanically polished using 400-, 600-, 800- and 1000-grit SiC paper and then electropolished for microscopic observation and nanoindentation testing.

A Zeiss Gemini 300 SEM (Jena, Germany) with an Oxford X-Max 80T EBSD system was used for the microstructure characterization. The results included the recrystallization grain size and area percentage of the recrystallization.

Nanoindentation testing was completed on a Hysitron TI950 nanoindentor under the force control mode. The maximum load was 5 mN. For each specimen, an array of 25 × 6 indents was made on the TD section, as shown in Figure 1a. The spacing between indents was 5 μm, which was 3 times longer than the typical indent size.

Quasi-static tension testing was carried out on an MTS Landmark testing system. The strain rate was approximately 5 × 10^−4^ s^−1^. The specimen geometry is shown in Figure 1b.

The fatigue crack growth testing was carried out on the same machine used for the tensile testing. The stress ratio was 0.1, with a fatigue frequency of 30 Hz. The single edge crack tension (SET) specimens were designed for the fatigue crack growth testing as shown in Figure 1c. At first, the fatigue crack at the notch root was prefabricated by the *K*-decreasing method for all specimens. Then, the fatigue crack propagation threshold and the fatigue crack growth rate were obtained using the constant force amplitude test. In order to eliminate the influence of the previous load in the fatigue pre-cracking, only those data obtained after the crack size increment of 5 or more times the plastic zone size under the pre-cracking load were considered valid data. During the fatigue testing, the fatigue crack lengths were measured by using the electric potential difference method [20]. The Zeiss Gemini 300 SEM was also used for fracture surface analysis and observation of the crack paths.

For investigation of the micro mechanisms of fatigue crack propagation, the in situ EBSD measurements were carried out during the fatigue crack growth testing. At first, the EBSD pictures were taken at the front areas of the crack tips of the specimens after the crack extension of 1~2 mm. The fatigue crack growth continued until the cracks extended beyond these areas. Finally, the EBSD pictures were taken at these same areas. By comparing EBSD maps from before and after the crack growth, the changes of the grain orientation and the kernel average misorientation (KAM) values would provide clues for discovering the mechanisms of crack growth.

## 3. Results

### 3.1. Microstructures

**Figure 2:**
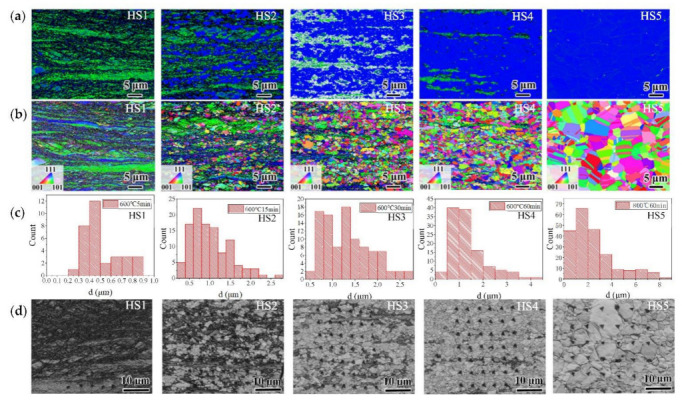
EBSD maps of the TD direction of the specimens with different levels of heterogeneity. From left to right are the HS1 to HS5 samples, respectively. (**a**) KAM maps. (**b**) Orientation maps. (**c**) Statistical diagrams of the sizes of the RGs. (**d**) Pattern quality maps of the TD direction of the specimens, with parts of the indentations.

The EBSD images of the TD sections of the specimens with heterogeneous microstructures are shown in Figure 2. It is clear that these microstructures are of the lamella type, which consists of hard, ultra-fine grains (UFGs) and soft, recrystallized grains (RGs). With increasing the annealing time from 5 min to 60 min, the morphology of the microstructures changed from deformed forms to partially recrystallized forms. The specimens annealed at 1073 K for 60 min were fully recrystallized. As shown in Table 1, the area percentage of recrystallization increased gradually from 10.2% to close to 100%.

The statistic sizes of the RGs are shown in Figure 2c. For the HS1 specimens, the sizes of the RGs were very fine, with mean value of less than 0.51 μm. For the HS2, HS3, and HS4 specimens, the mean values of the sizes of the RGs increased with the increase in recrystallization from 1.02 to 1.37 μm. For the HS5 specimens, which were fully recrystallized, the sizes of the RGs dramatically increased to a maximum size of 8–9 μm.

### 3.2. Tensile Stress–Strain Curves and Nanoindentation Hardness

**Figure 3:**
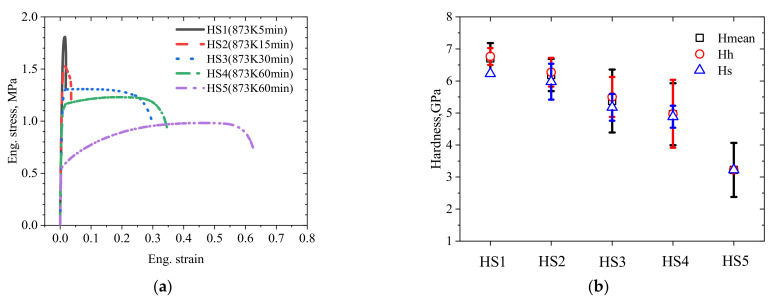
Mechanical properties of the CrCoNi MEAs subjected to different heat treatments. (**a**) Tensile stress–strain curves. (**b**) Statistical data of the indentation hardness at the TD directions of the specimens.

The tensile stress–strain curves of the specimens with heterogeneous microstructures are shown in Figure 3a. It is clear that the yield strengths decreased with the increase in recrystallization and the uniform elongations increased with the increase in recrystallization. For HS1 and HS2, the curves had a high yield strength and very low ductility. For HS3, the curve had a uniform elongation (*ε*_ue_) of 13.4%, without any strain hardening. For HS4, it had relatively larger uniform elongation of 23.1% and a higher yield strength. HS5 had the largest uniform elongation of 49.5% and the lowest yield strength of all the specimens.

Parts of the indentations of the specimens with heterogeneous microstructures are shown in Figure 2d. As seen in the KAM maps and the pattern quality maps, the indentations on the deformed areas and the recrystallization areas are very clearly distinguished. This means that the hardness of all the indentations (the hardness of the indentations in the hard areas or in the soft areas) could be analyzed separately.

The statistical data of these measures of hardness are shown in Table 1 and Figure 3b. As shown in Figure 3b, it is clear that the hardness of all the indentations (*H*_mean_) decreases with the increase in recrystallization as changes of the tensile strength. However, for all specimens, the mean hardness in hard areas (*H*_h_) was slightly higher than those in soft areas (*H*_s_), and these differences are not immediately obvious. This may be because the fine RGs induced higher levels of strength, as described in the Hall–Petch relationship.

### 3.3. Fatigue Crack Propagation

**Figure 4:**
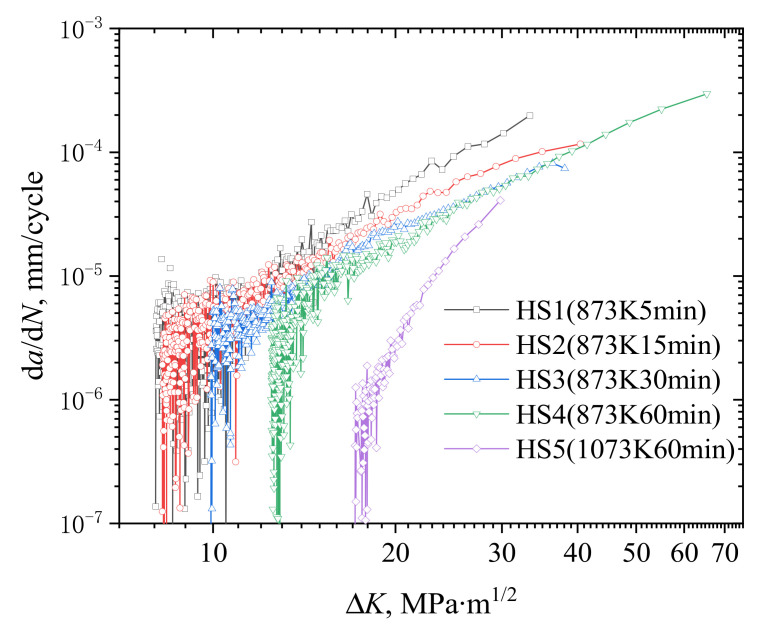
d*a*/d*N*-Δ*K* curves of the specimens with different heterogeneous microstructures.

The d*a*/d*N*-Δ*K* curves of the specimens with different heterostructured materials are shown in Figure 4. In this diagram, it is clear that the thresholds of the fatigue crack growth increased with the increase in the recrystallization, while the fatigue crack growth rate decreased. For all specimens, the fatigue crack growth rates fluctuated in the near-threshold region, which could be attributed to the inhomogeneous microstructures. For HS1 and HS2, the fatigue crack growth thresholds Δ*K*_th_ were approximately 8.5 MPam^1/2^. For HS3, the threshold increased to 9.9 MPam^1/2^. For HS4 and HS5, the Δ*K*_th_ increased to 12.9 MPam^1/2^ and 17.9 MPam^1/2^, respectively.

Compared with the other parameters listed in Table 1, it can be found that with the increase in recrystallization temperature and time, Δ*K*_th_ increased synchronously with the size of the RGs, the area percentage of recrystallization, and the uniform elongation, but it was opposite to the change in yield strength, tensile strength, and indentation hardness. The relationship between these parameters and the degree of heterogeneity are discussed in the next section.

**Figure 5:**
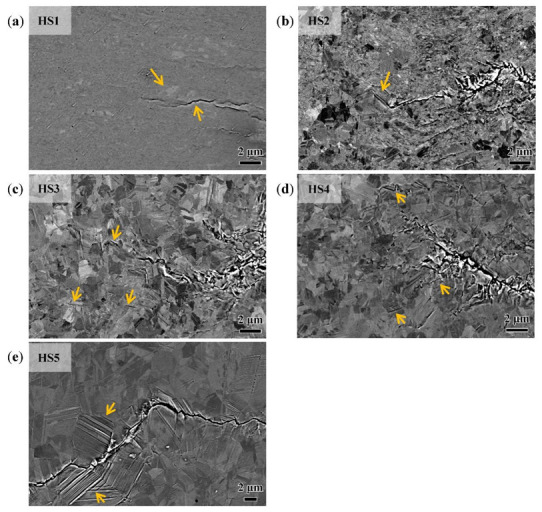
BSE images of the fatigue crack growing paths on the different heterostructured specimens. (**a**) HS1, fatigue crack bypassed RG when the crack tip met it (indicated by arrows). (**b**) HS2, fatigue crack grew alone the slip plane of the RG when the crack tip met it (indicated by arrow). (**c**) HS3, (**d**) HS4, and (**e**) HS5. Isolated microcracks appeared along the twin boundaries (indicated by arrows in (**c**–**e**).

The Zeiss Gimin300 SEM’s back-scatter electron (BSE) image has a contrast of crystal orientation that can be used to discover the mechanisms of crack propagation. As shown in Figure 5a–d, for all the specimens of different heterogeneous microstructures, the recrystallized grains with sizes of 1–2 μm are surrounded by the deformed areas. In the HS1 specimens, which had the lowest volume percentage of recrystallization, the cracks propagated in the deformed areas with shear models. At the crack tip, there were many isolated micro cracks distributed along the orientation of the maximum shear stress, and cracks would change direction when meeting a recrystallization grain, as indicated by the arrow shown in Figure 5a. For HS2, which had 24.0% RGs, the fatigue crack propagation behavior was very similar to that of HS1. However, as indicated by the arrow shown in Figure 5b, it is clear that the crack grew alone the slip plane of the RG when the crack tip met it, and there are many deformation bands that appeared in other adjacent RGs. For the other specimens with a high percentage of recrystallization, these deformation bands were more frequently seen in the neighboring grains, and there were some isolated microcracks that appeared along the twin boundaries at the adjacent area, as indicated by the arrows shown in Figure 5c–e. These mean that the crystal orientation of the RGs would induce the crack deflection and branching, and thus have quite an effect on the fatigue crack propagation thresholds.

**Figure 6:**
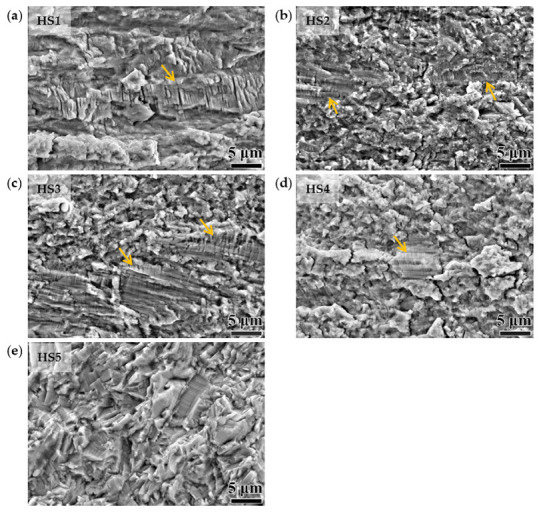
SE images of fracture surfaces of the different heterostructured specimens. (**a**) HS1, (**b**) HS2, (**c**) HS3, (**d**) HS4, and (**e**) HS5. The lamellar structures of the hard zones with fatigue striation were indicated by arrows in (**a**–**d**).

Photos of the fracture surface are shown in Figure 6. Corresponding to the image of the crack growing path, there are two morphological features on the fracture surface. One is of the lamellar structure of the hard zones with fatigue striation, as indicated by the arrows in Figure 6. Another one is of the granular structure of the RGs, which is in accordance with the cleavage fracture in Figure 5. With the increase in the area percentage of the recrystallization, the area of the granular structure increased and the surface became rougher. HS5′s fracture surface was full of cleavage fractures, as shown in Figure 6e.

**Figure 7:**
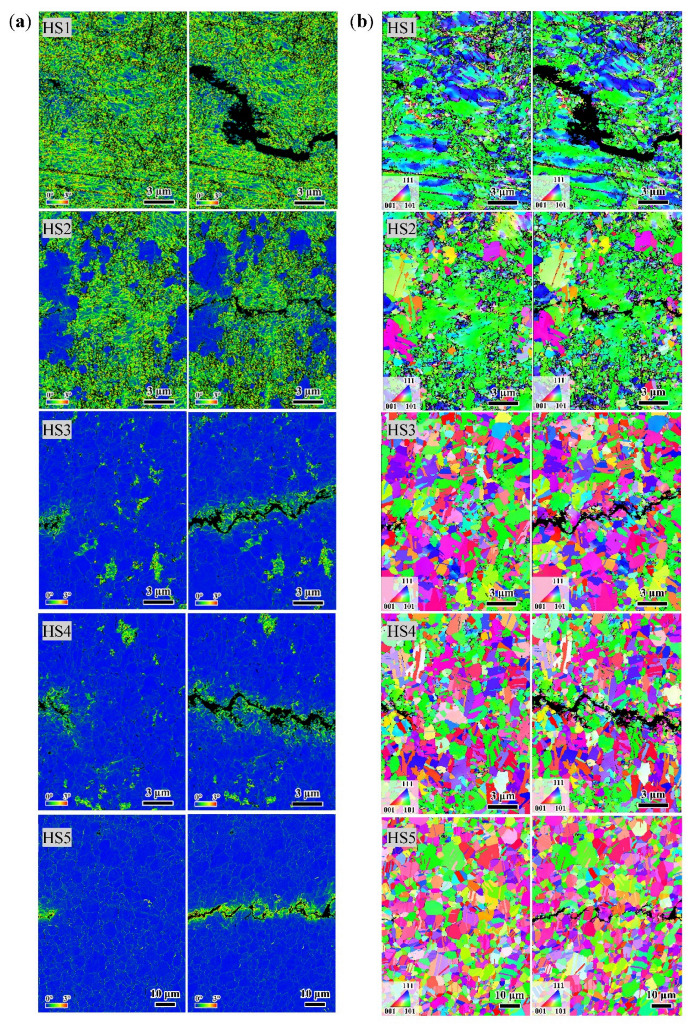
The EBSD maps of the in situ fatigue crack propagation. For each specimen, the left map was acquired at the front of the crack tip and the right map was acquired at the same site, but after crack growth. (**a**) KAM maps. (**b**) Orientation maps.

The results of in situ EBSD measurements are shown in Figure 7. Figure 7a shows a diagram of all the KAM maps, and Figure 7b shows a diagram of all the orientation maps. For each diagram, the left column shows maps of all the specimens acquired at the crack tips and the right column shows maps of all the specimens acquired at the same sites after fatigue crack growth. As shown in Figure 7b, the crack paths all zigzag. For HS1 and HS2, these crack deflections appeared at the boundary between the deformed areas and the recrystallization areas. For HS3, HS4, and HS5, these deflections were related to the change in grain orientation along the crack growth path. These were consistent with the results of the BSE images in Figure 5. However, except for the nearest neighbor grains along the crack, no obvious difference was observed in the crack tip region before or after crack propagation. No obvious morphological changes were observed, and no deformation twins and slip bands, as shown in the BSE images, were observed. In addition, for all specimens, except for the nearest neighbor regions along the crack, no obvious changes of the KAM values were observed in the crack tip region before or after crack propagation. The widths of the regions with increased KAM values were about 1–2 times the grain sizes of the specimens, as shown in Figure 7a.

## 4. Discussion

### 4.1. Quantitative Characterization of the Heterogeneous Microstructures

For heterostructured materials, how to quantify their heterostructured levels is very important. In general, it can be considered from the microstructural morphology and the difference of the micromechanical properties.

**Figure 8:**
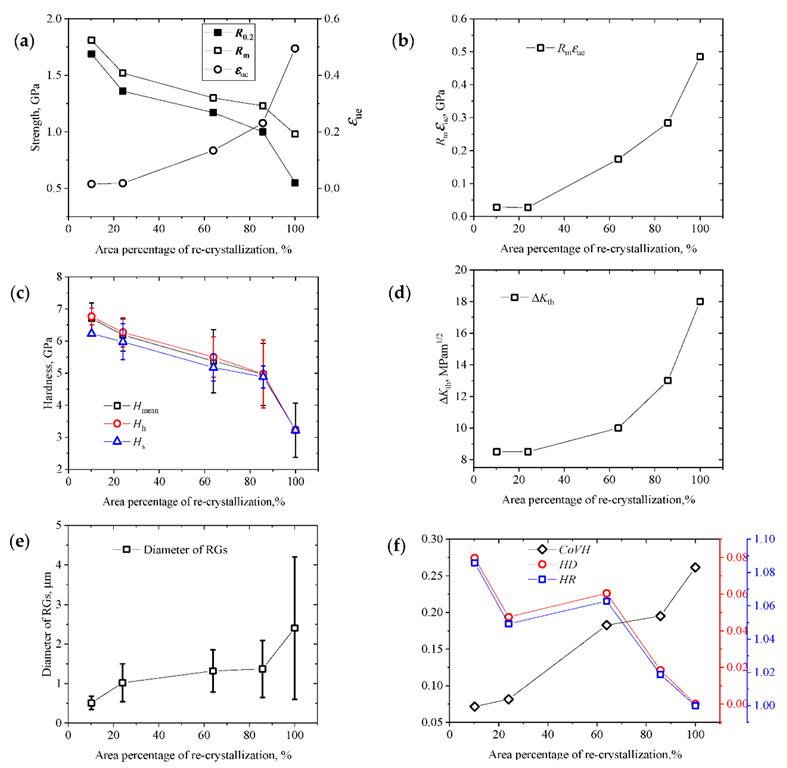
Experimental data versus the area percentage of recrystallization. (**a**) Tensile strengths and uniform elongations. (**b**) Product of the strength and uniform elongation. (**c**) Nanoindentation hardness. (**d**) Fatigue crack propagation thresholds. (**e**) Diameters of the RGs. (**f**) Hardness parameters for quantifying the heterostructured level.

Considering the microstructural morphology, the area percentage of the recrystallization is the simplest parameter for characterization of the heterostructured level. A 50% area percentage of the recrystallization refers to the highest level of the heterostructure. As shown in Figure 8, the tensile strength and hardness decreased with the increase in the area percentage of the recrystallization, and the uniform elongation, the product of strength and uniform elongation, the fatigue crack propagation threshold, and the diameter of the RGs were increased with the increase in the area percentage of recrystallization. It appears that the heterostructured level has no obvious effect on these experimental results. For HS4, which had a related higher strength and higher uniform elongation as a typical example of heterostructured material, its area percentage of recrystallization was 85.8% which indicates a lower heteostructured level. For HS1, its area percentage of recrystallization was only 10.2%. Its heteostructured level was even lower than HS4, but its uniform elongation was very low. For HS3, its area percentage of recrystallization was 63.9%, and it had a higher heteostructured level but a lower level of strength and uniform elongation than HS4. Therefore, considering only the microstructural morphology was not enough to explain the effects of the heteostructured level on the mechanical properties.

Considering the differences of the mechanical properties of the hard zones and the soft zones, parameters including the hardness ratios of the hard and soft regions (*HR*), hardness difference of the hard and soft regions (*HD*), and the coefficient of variation of all hardness (*CoVH*) are all candidate parameters for quantifying the heterostructured levels. The definitions of these parameters are shown as follows:

(1) $$HR=\frac{H_{h}}{H_{s}}$$

(2) $$HD=\frac{H_{h}-H_{s}}{H_{\mathrm{mean}}}$$

(3) $$CoVH=\frac{Sd_{H}}{H_{\mathrm{mean}}}$$

Here, *H*_h_ is the mean value of hardness of the hard zone, *H*_s_ is the mean value of hardness of the soft zone, *H*_mean_ is the mean value of all hardness, and *Sd*_H_ is the standard deviation of all hardness.

As shown in Figure 8f, the curves of the *HR* and *HD* versus the area percentage of recrystallization are very similar, which is likely because of the small difference in the hardness between the hard regions and soft regions for all specimens in this study. According to the values of the *HR* or *HD*, HS1, HS2, and HS3 have related higher heterostructured levels. Here, HS1 has the highest heterostructured level, which is in contradiction to the morphological observation results. From this diagram, it appears that specimens with higher heterostructured levels did not have good combinations of strength and ductility.

Comparing the area percentage of recrystallization and the *HR* or *HD*, the former only considers the microstructural morphology, and the latter two only consider the difference in mechanical properties between the hard and soft zones. Either one is incomplete. A better parameter for the heterostructured level must include a comprehensive consideration of the microstructural morphology and the difference in mechanical properties. For *CoVH*, its value comes from the calculation of all hardnesses which are evenly distributed in the TD section, and so, it includes both information about the microstructural morphology and the difference in the mechanical properties.

Interestingly, contrary to the *HR* and *HD*, the values of *CoVH* increase with the increase in the area percentage of recrystallization. This was very similar to the changes of ductility and the fatigue crack propagation threshold. According to the values of the *CoVH*, HS5, which was fully recrystallized, had the highest heterostructured level.

In summary, quantifying the heterostructured levels is very complex. The values defined by different points varied greatly, which would induce the opposite conclusion about the effect of the heterostructured levels. Therefore, it is difficult to explain the mechanical properties of heterostructured materials by these phenomenological methods.

In general, for heterostructured materials with mixed soft zones and hard zones, dislocations would be the first to emit in the soft zones at the initial stage of a deformation. Then, these dislocations would pile up at the boundary of the hard zone and soft zone. This would indicate that the strength of the hard zone determines the strength of the heterostructured materials. On the other hand, the uniform elongation is determined by the strain-hardening capacity of the conventional dislocation hardening and hetero-deformation induced (HDI) hardening, which are related to the area percentage of soft zones and the density of the boundary of the hard and soft zones [5]. In the present study, all specimens were produced by recrystallization annealing. The hardness and the area percentage of the hard zones both decreased with an increase in the area percentage of the recrystallization, which means that the effects of the microstructural morphology and the difference of the micromechanical properties were mixed. For this reason, building a universal parameter of the heterostructured levels is difficult.

### 4.2. Mechanism of Fatigue Crack Propagation for MEAs with Different Heterogeneous Microstructures

Integrating the results of BSE images, EBSD maps, and fracture surface analysis, it was observed that the crack growth mode of the CrCoNi alloys with different heterogeneous microstructures change with the microstructure. In general, fatigue cracks pass directly through deformed areas. The direction of the crack path is almost perpendicular to the loading direction. When a crack tip meets an RG, it would grow along the twin boundary or slip band, which would induce the crack path deflection with the orientation of the RGs. This deflection changes the crack from mode I to mode I + II mixed-type, which would decrease the stress intensity factor at the crack tips. Meanwhile, this change of crack mode would also bring the roughness-induced crack closure effect, which plays a major role near the fatigue crack propagation threshold. This phenomenon becomes more obvious as the area percentage increases.

In this investigation, we noted that the size of the RGs also increases with the increase in the area percentage of the recrystallization, and it is necessary to consider both the effects of grain size and the area percentage of the recrystallization on the fatigue crack propagation threshold. For HS1, HS2, and HS3, although the area percentage of the recrystallization increased from 10.2% to 63.9%, as shown in Table 1, the increase in the grain size of the RGs was small. Therefore, the roughness-induced crack closure effect was not obvious and there was little increase in the value of the fatigue crack propagation threshold. However, for HS5, with its maximum grain size of 10 μm, the effect of this crack deflection was a significant increase. The fatigue crack propagation threshold increased to 17.9 MPam^1/2^, which is more than twice as much as that of HS1 and HS2.

In addition, attention should be paid to the influence of a specimen’s strength. In general, the effect of strength on the crack propagation threshold is actually attributed to the grain size of the specimen. According to the Hall–Petch relation, the variation of the strength actually reflects the change in the grain size. In this paper, although most of the microstructures are partially recrystallized, the Hall–Petch relation is still valid for the size of the RGs.

In the paper written by Zerbst [17], the size of a cyclic plastic zone was determined to be approximately one tenth of the size of the plastic zone under plane strain conditions, as follows [21]:

(4) $$r_{\mathrm{cp}}\approx \frac{1}{30\pi }{\left(\frac{K_{I}}{\sigma_{y}}\right)}^{2}$$

Here, *K*_I_ is the stress intensity factor for a mode I crack and *σ*_y_ is the yield strength.

According to Equation (4), the cyclic plastic zone sizes of all the specimens at the fatigue crack propagation threshold and at the sites of the in situ testing were calculated. The results are shown in Table 2. It can be clearly found from the results that the cyclic plastic zone sizes are equal to the sizes of the RGs in the order of magnitude. This result supports that the stress intensity factor range at the crack tip is the fatigue crack propagation threshold Δ*K*_th_ when the plastic zone size equals the grain size of the specimen. Here, the plastic zone size is the size of the cyclic plastic zone rather than the plastic zone under single loading.

As shown in Figure 7, the results shown in the EBSD orientation maps and KAM maps indicate that only the neighboring grains along the crack path are affected by the crack growth, which is consistent with the results of the sizes of the cyclic plastic zone shown in Table 2. This indicates that the larger lattice distortion only appeared in the cyclic plastic zone, rather than in the plastic zone under the single loading. In reality, no morphology changes and lattice distortions appeared in the plastic zone of single loading. This could mean that the dislocation motion was recovered under the cyclic loading in this region. However, the grains in the cyclic plastic zone accumulated a large plastic strain with the increase in loading cycles. According to the sizes of the cyclic plastic zone and the fatigue crack growth rate, the number of loading cycles for every site in the cyclic plastic zone could be estimated. For every specimen in Figure 7, the numbers are from about 100 to 10,000, respectively.

Another interesting phenomenon is related to HS4, which had a good combination of high tensile strength and a large uniform ductility. Yang et al. [22] observed that the grain structure of this specimen possessed three levels of grains with obviously different sizes: MGs, UFGs, and NGs. They called it a highly heterogeneous grain structure (HGS). In tensile testing, they found the in situ production of new nanograins at grain boundaries throughout the tensile straining. This grain refinement made the grain structure even more heterogeneous, which induced additional HDI strengthening and hardening. However, in this investigation, no deformation-induced grain refinement was observed in the plastic zone at the crack tip before the crack growth. This indicated that the movement of dislocations, deformation twinning, and stacking fault might be recovered under cyclic loading, which suppressed the formation and growth of the nanocrystalline, and which is different than under tensile loading.

There are some nanocrystalline grains that appeared along the crack growth paths after the crack propagation, as shown in Figure 7b. This phenomenon was reported in many investigations on the very high cycle fatigue (VHCF) of the metals [23,24,25,26,27,28]. Usually, these nanocrystalline grains appeared at the fracture surface in the source region of the fatigue crack. Hong et al. [23,29] believed that this was a phenomenon formed by the repeated extrusion of the crack surface, and they proposed an NPC model to explain this phenomenon. However, in present study, the number of repeated extrusions in the cyclic plastic zone is a few orders of magnitude smaller than that in a VHCF. Therefore, these observed nanocrystalline grains could be the result of grain fragmentation at the moment of crack growth.

## 5. Conclusions

Five sets of specimens of CrCoNi MEAs with different heterostructured levels were produced by cold-rolling and recrystallization annealing. Based on the measurements of the EBSD and nanoindentor, the quantitative characterization of the heterogeneous microstructures was discussed from the aspects of micro-morphology and micromechanical properties. Fatigue crack growth testing was carried out, and the fatigue crack growth behaviors and mechanisms were studied using EBSD in situ testing, BSE crack path observations, and fracture analyses. The conclusions are as follows:

1. From the aspects of micro-morphology and micromechanical properties, the area percentage of recrystallization, hardness ratio of the hard and soft regions (*HR*), hardness difference of the hard and soft regions (*HD*), and coefficient of variation of all hardness (*CoVH*) are all candidate parameters for quantifying the heterostructured levels. When the parameters defined by different methods were associated with performance, different results were obtained.
2. The damage of the fatigue crack growth mainly appeared in the cyclic plastic zone at the crack tips, which was equal to the size of the RGs in the order of magnitude. In this region, there are slip bands, deformation twinning, and some micro cracks along the twin boundary. There were no obvious changes in micro-morphology, orientation and lattice distortion observed outside the cyclic plastic zone.
3. The fatigue crack growth path was deflected due to the orientation of grains at the crack tip, which changed the crack to a mixed-mode, decreased the stress intensity factor, and induced the roughness-induced crack closure effect. A larger recrystallization percentage and recrystallized grain size will increase the fatigue crack propagation threshold and decrease the crack growth rate.

## Figures and Tables

**Table 1:** The statistical results of the microstructures and mechanical properties.

Specimen	Annealing Process	Sizes of RGs μm	Area Percentage of Recrystallization	*H*_mean_ GPa	*H*_h_ GPa	*H*_s_ GPa	*R*_0.2_ GPa	*ε*_ue_	*R*_m_ GPa
HS1	600 °C 5 min	0.51 ± 0.17	10.2%	6.70	6.76	6.23	1.69	0.0154	1.81
HS2	600 °C 15 min	1.02 ± 0.48	24.0%	6.19	6.27	5.98	1.36	0.0181	1.52
HS3	600 °C 30 min	1.32 ± 0.53	63.9%	5.37	5.50	5.18	1.17	0.134	1.30
HS4	600 °C 60 min	1.37 ± 0.72	85.8%	4.96	4.98	4.88	1.00	0.231	1.23
HS5	800 °C 60 min	2.4 ± 1.8	100%	3.22	3.22	3.22	0.55	0.495	0.98

**Table 2:** The cyclic plastic zone sizes of all the specimens at Δ*K*_th_ and the sites of the in situ testing.

Specimen	*R*_0.2_, GPa	Δ*K*_th_, MPam^1/2^	Cyclic Plastic Zone Size at Δ*K*_th_, μm	Sizes of the RGs, μm	ΔK at the In Situ Testing, MPam^1/2^	Cyclic Plastic Zone Size at the In Situ Testing, μm
HS1	1.69	8.5	0.33	0.51 ± 0.17	13.0	0.77
HS2	1.36	8.5	0.51	1.02 ± 0.48	13.1	1.21
HS3	1.17	9.9	0.94	1.32 ± 0.53	13.7	1.81
HS4	1	12.9	2.18	1.37 ± 0.72	16.5	3.58
HS5	0.55	17.9	13.9	2.4 ± 1.8	18.6	15.0

## Data Availability

Not applicable.
